# Stress and Heart Rate Variability during University Final Examination among Lebanese Students

**DOI:** 10.3390/bs9010003

**Published:** 2018-12-27

**Authors:** Sabah Hammoud, Rita Karam, Rabih Mourad, Iman Saad, Mazen Kurdi

**Affiliations:** Laboratory of Experimental and Clinical Pharmacology, Department of Chemistry and Biochemistry, Faculty of Sciences, Section 1, Lebanese University, Rafic Hariri Educational Campus, P.O. Box 6573/14 Hadath, Lebanon; sabah.hammoud2@gmail.com (S.H.); ritakmouawad@hotmail.com (R.K.); rabih.mourad@hotmail.com (R.M.); isaad@ul.edu.lb (I.S.)

**Keywords:** electrocardiography, heart rate, autonomic nervous system, stress, university

## Abstract

Real-life stressors, such as university examination, cause an increase in sympathetic activity of the nervous system innervating the heart, and thus an increase in heart rate (HR). Our study aimed to detect changes in heart rate variability (HRV) during different stages of an exam in a group of 90 healthy university students (30 males and 60 females), over 4 h of monitoring divided into 1 h before, 2 h during, and 1 h after the examination. HRV was significantly highest after the exam, indicating release from stress, as compared to before and during the examination when stress was observable. Undergraduate students in different academic years did not differ in terms of stress, indicating the absence of adaptation to exam procedures. However, HR and R-R interval after the exam showed significant difference between first year undergraduate studies and first year of a graduate program, indicating a higher degree of confidence in graduate students. Results also suggest that HRV in females is significantly lower than that in males before and after examination, despite men having greater sympathetic input. In conclusion, the results of our novel study assessing stress in real-time examination show important gender differences, and lack of adaptation with academic study year.

## 1. Introduction

Heart rate variability (HRV) is a promising tool for assessing cardiovascular health and the degree of severity of cardiovascular diseases [1]. It is a non-invasive and valuable method to assess autonomic functioning of the heart from a simple electrocardiogram (ECG) recording. HRV is manifested by the variability in R-R intervals of successive heart beats, which reflects the sympathetic and parasympathetic activity of the autonomic nervous system (ANS) innervating the heart. ANS activity regulates the cardiac cycle of contraction in response to various changes and demands of the body. In the presence of cardiovascular disorders or one of their risk factors such as stress, the sympathetic nervous system predominates in the heart over the parasympathetic one, leading to increased heart rate (HR) and thus a decrease in the beat-beat variability [2].

HRV is affected by various factors including physical activity, such as running or practicing yoga, where subjects who exercise regularly possess lower HR and present an increase in HRV [3]; exercise has also shown to slightly reduce the sympatho-vagal activity, inferred through the low frequency (LF)/high frequency (HF) ratio component of HRV [4]. This represents some kind of adaptation of the heart to external stimulators and its response to them. Furthermore, other lifestyle factors, such as smoking and caffeine intake, may affect HRV when studying the heart response to a certain stressor stimulant. As such, people with a worrying trait who consume more caffeine or smoke regularly show higher HR and lower variability in their heartbeat pattern [5].

Several studies have been done to assess the effect of mental stress on HRV as well, mainly observing the variability during the examination time for healthy university students. Upon a mental stressor, the ANS balance of the body is disrupted, leading to an increase in the sympathetic activity and a decrease in the vagal activity of the heart [6]. In examination conditions, students present lower beat-beat variability compared to normal non-academic situations [7,8,9], and subsequently present the highest HR during the examination [10]. The frequency component of HRV are the main affected parameters, where LF increases and HF decreases, leading to a significant increase in their corresponding ratio [6,11]; this increase in the sympathetic activity is independent of the respiratory changes found in the presence of a stressor [11], where the degree of this variation depends on level of anxiety and worry while doing the assigned mental stress task [6]. Random studies covered short-term monitoring of 5 min before and after an exam [8,12,13] or 24 h monitoring during regular academic activities [14]. 

The Lebanese University is the only public university in Lebanon and includes approximately 50% of all university students in Lebanon. Students at the Lebanese University generally pass through a stressful period during the examination time; the main goal of this research was to study the changes in HR and HRV parameters, which reflect stress levels among university students just before, during, and after the exam takes place; subsequently, another aim was to assess such HRV changes based on gender. 

Moreover, it was of interest to compare the ANS response, represented by HRV parameters, among students in different academic years and to study the reliability of their stress self-assessment. To our knowledge, this study is the first to detect HRV parameters for students conducting an official university examination, and it will reveal the correct outcome of stress induced by examination. Considering that students undergo such written examination procedures regularly over a period of 3 to 5 years during their studies, it is postulated that exam-induced stress could affect the heart health.

## 2. Material and Methods

### 2.1. Study Design and Participants

The study protocol was approved by the institutional review board committee represented by the Council of the Faculty of Sciences at the Lebanese University, Section I. A random sample of 90 healthy students [30 males (33.33%) and 60 females (66.67%)] whose ages range from 18 to 23 years (mean age 20.57 ± 1.26 years) were enrolled in the study. Participants were pursuing their studies at the Faculty of Sciences—Section 1 between the first and the third academic year of undergraduate program, or enrolled in the first academic year of graduate program; subjects were distributed as 25 students in the first academic year (28%), 18 students in the second year (20%), 39 students in the third year (43%), and 8 students in the first year graduate program (9%). Study participants had to be healthy, disease free, where included subjects were not under treatment with any drug or supplements during the examination period. Exclusion criteria comprised individuals with cardiovascular diseases, subjects suffering from severe tachycardia, and other disorders such as diabetes and hypertension. All participants signed an informed consent form prior to their participation, where a written and verbal explanation of the study protocol was provided. Subjects were coded using application number to confirm confidentiality and to avoid bias during analysis.

### 2.2. Study Procedure

**Figure 1:**
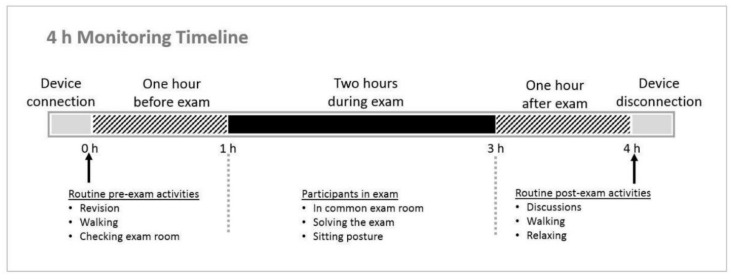
Illustration of study protocol during final examination.

Participants were asked to be present at the laboratory one hour in advance of their examination time. Students first signed the informed consent, then a one channel monitoring device (CardioDiagnostics cardiac monitoring devices) was connected by the use of two-lead cable and Ambu^®^ WhiteSensor WS (Copenhagen, Denmark) electrodes, which were placed on the right chest and left abdomen to record ECG throughout the procedure. Monitoring was done for approximately 4 h, which covered one hour before the exam, 2 h during the exam, and one hour after the exam was over (±15 min); after that, the device was disconnected (Figure 1). Additionally, a questionnaire about lifestyle during the examination period was completed before the exam took place. Also, students were requested to self-evaluate their stress levels before, during and after examination as not stressed, moderately stressed, or highly stressed. 

### 2.3. HRV Analysis

By means of Kubios HRV version 2.2 software (Kubios, Finland), the recorded ECG data were analyzed at a sampling rate of 250 Hz, and several HRV parameters were assessed, divided into time domain parameters, frequency domain factors, and the non-linear domain analysis (Table 1). ECG analysis was visually inspected to diagnose for any abnormal signaling, during which manual selection of QRS complexes was done where necessary, along with low to medium artifact corrections to optimize HRV outcomes. 

Timeline of the 4 h monitoring procedure, displaying the time of device connection one hour prior to examination period. Monitoring covered one hour before exam while doing routine pre-exam activities, 2 h during an exam in routine academic conditions, and one hour after exam while doing routine post-exam activities; after 4 h, the devices were disconnected.

Time domain analysis include average R-R interval [ms], and SDNN [ms], its corresponding standard deviation. Also, average HR was evaluated and expressed as beat per minute [bpm], where a normal resting heart rate can vary between 60 and 90 bpm. The parasympathetic activity of the heart was analyzed through RMSSD [ms], the root mean square difference of successive R-R intervals, where an increased RMSSD represents a predominance of the parasympathetic system in the heart during the analyzed time interval. pNN50 [%] is another factor in time domain analysis, which is the percentage of successive R-R intervals that differ by more than 50 ms. An increase in SDNN, RMSSD and pNN50 leads to an increase in HRV and are usually accompanied with lower HR values.

With regard frequency domain analysis, it is estimated using parametric autoregressive (AR) modeling—based methods; it is analyzed in terms of LF (bands belonging to 0.04–0.15 Hz) and HF (bands belonging to 0.15–0.4 Hz) factors, which reflect sympathetic and parasympathetic activity of the heart, respectively; the latter mentioned components are demonstrated in normalized values (%), to be better compared between individuals [15]. A ratio of these 2 activities, the LF/HF ratio, determines the balance in ANS activity of the heart, and the predominance of the sympathetic or parasympathetic activity acting on it. As LF and LF/HF ratio increase, they indicate an increase in the activity of the heart, and thus may reflect the presence of stress conditions.

Finally, HRV can be also evaluated through non-linear analysis, mainly presented by Poincare plot. The standard deviation across the short axis (SD1 [ms]) and long axis (SD2 [ms]) of this plot estimates the short-term and long-term HRV, respectively. Therefore, a higher HRV is demonstrated by a raise in SD1 and a decline in SD2.

Data analysis covered 30 min interval before, during and after taking the exam. These intervals were chosen based on the participants’ report of the specific time entering and leaving the examination place, and the exact time of starting and ending the exam; a minimum of 15 min was left as border time interval between different phases of the examination to exclude transition interaction between different conditions.

### 2.4. Statistical Analysis

SPSS was used for statistical analysis. Values were displayed as mean ± SEM (standard error mean). Differences among several groups were analyzed using one-way analysis of variance (ANOVA) to test interaction between and within subject factors, followed by post-hoc analysis of Tukey to evaluate the significance of difference among means when at least 3 groups were involved. ANOVA of repeated measures was used to analyze the HRV changes in different phases of examination, and two-way ANOVA and three-way ANOVA were performed to study possible factor-factor interactions. Additionally, Chi-squared test was used to determine the presence of any association between categorical parameters among the included sample. *P* values less than or equal to 0.05 were considered to be statistically significant.

## 3. Results

### 3.1. HRV in Different Exam Stages

Before the exam, students presented significantly higher HR (110.93 bpm), and lower R-R interval (553.44 ms) compared to the period of exam (*P* < 0.001), as well as significantly higher SDNN (72.60 ms) compared to during the examination phase (54.03 ms); while no significant difference was found in other time domain parameters. However, after the exam, SDNN significantly increased compared to before and during the exam (89.78 ms, *P* < 0.001), RMSSD significantly increased compared to the 2 hour period during the exam (46.55 ms, *P* = 0.05), and pNN50 was found to be significantly increased compared to before the exam based on post-hoc analysis (13.01%, *P* = 0.046). These changes denote an increase in the parasympathetic input of the ANS, and a decrease in the sympathetic one, after examination. Concerning frequency domain, LF and HF components were significantly higher during the exam (LF: 36.81%, HF: 17.09%) compared to before and after the examination time (*P* < 0.001), with no significant change in their corresponding ratio, which we interpret to mean that the highest activity of the ANS is present during examination. Regarding non-linear domain analysis factors, SD1 (32.66 ms) and SD2 (122.14 ms) were significantly higher after exam compared to before exam (*P* = 0.001) and to before and after examination (*P* < 0.001) respectively (Table 2). All these findings together are reflective of the dynamic changes in the physiology of the heart and change in stress levels throughout the testing procedure.

### 3.2. HRV among Students in Different Academic Years

**Figure 2:**
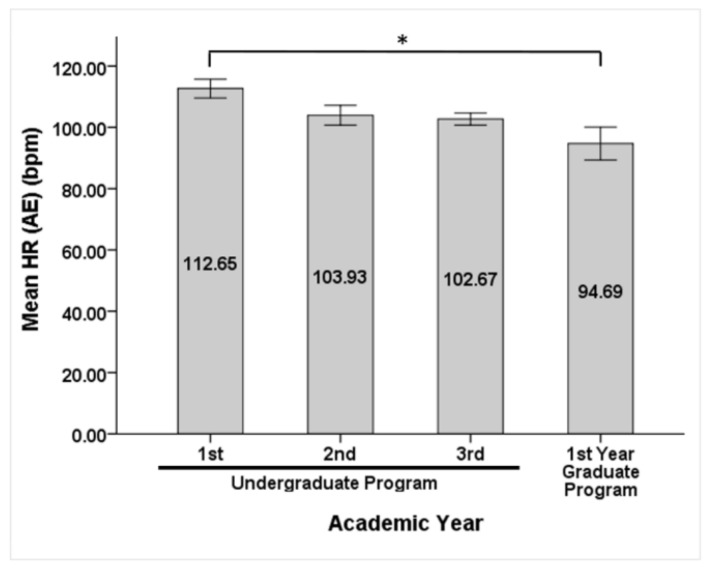
R-R and HR variation among different academic years after examination.

Students in the first year of the graduate program, (9% of participants) showed significantly higher R-R interval values (663.66 ± 112.66 ms, *P* = 0.007) and significantly lower HR (94.68 ± 15.2 bpm, *P* = 0.005) only in the 1 h interval period after exam compared to students in the first year of their undergraduate studies (28% of participants; R-R: 556.13 ± 76.17 ms and HR: 112.64 ± 15.46 bpm) (Figure 2). Other time, frequency and non-linear domain parameters were not significantly changed between students in different academic year (data not shown), suggesting that there is no difference in ANS activity innervating the heart, and stress levels are the same among students in different academic years. 

Bar graph showing mean HR (bpm) among students in different academic years after examination (error bars ± SEM); * represent significant difference between first- and fourth-year students with *P*-value ≤ 0.05.

### 3.3. HRV Gender Considerations

**Figure 3:**
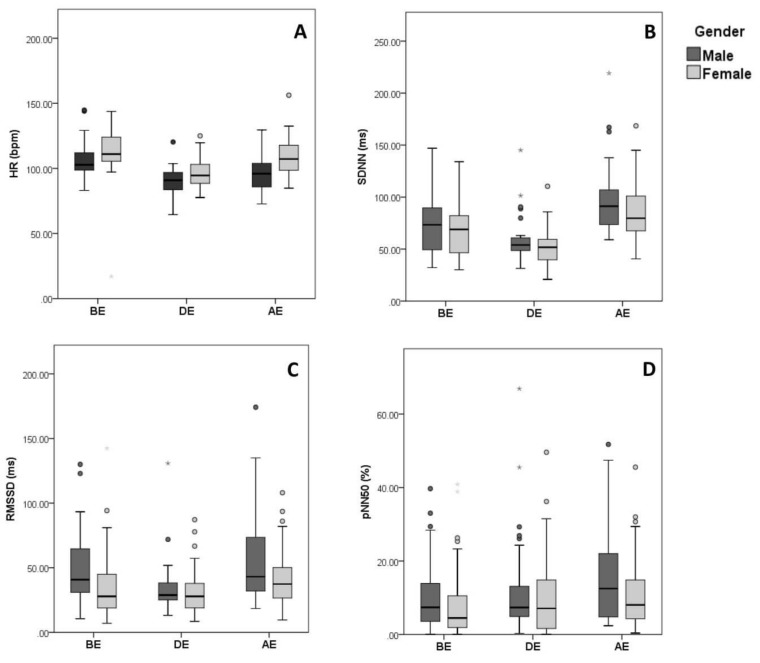
Data distribution of HRV time domain parameters based on gender, (**A**) HR, (**B**) SDNN, (**C**) RMSSD, (**D**) pNN50.

**Figure 4:**
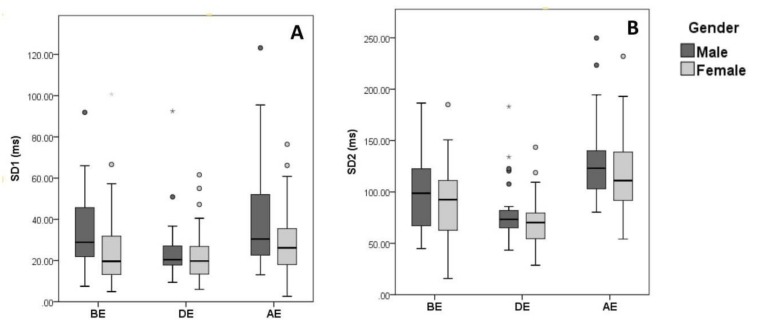
Data distribution of HRV non-linear parameters based on gender, (**A**) SD1, (**B**) SD2.

**Figure 5:**
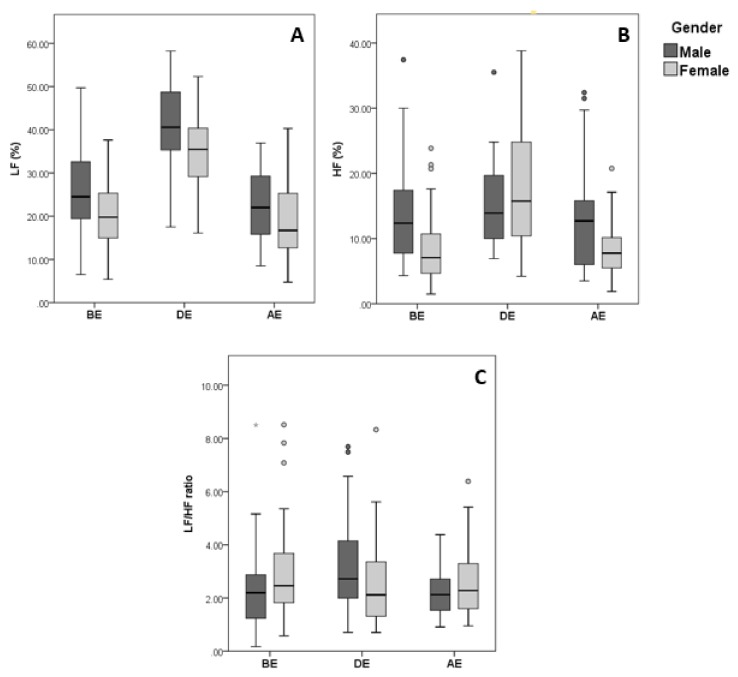
Data distribution of HRV frequency domain parameters based on gender, (**A**) LF, (**B**) HF, (**C**) LF/HF ratio.

Throughout the 3 phases of the examination, females possessed significantly higher HR than males (*P* = 0.05 BE, *P* = 0.018 DE, and *P* < 0.001 AE). This observation was accompanied by significantly lower HRV in females before and during examination, where males presented higher RMSSD (*P* = 0.009) and SD1 (*P* = 0.014) before exam, and higher pNN50 (*P* = 0.046), RMSSD (*P* = 0.009) and SD1 (*P* = 0.008) after exam is over (Figure 3 and Figure 4). However, during the exam, none of the time domain (other than HR) and non-linear domain parameters showed a significant difference between genders. Additionally, males showed significantly higher sympathetic activity as shown by the frequency component LF (*P* = 0.007, 0.003 and 0.022 BE, DE, and AE, respectively) (Figure 5). A significant change in LF/HF ratio was observed only during examination (*P* = 0.050) (Table 3). Thus, females exhibited more stress than males when compared in the prior and after the occurrence of a mental stressor, but they tend to stress similarly while the mental stressor is taking place.

Interestingly, three-way ANOVA analysis revealed no significant interaction between the effect of examination phase, gender of participant, and the academic year in which they are enrolled (*P* > 0.05).

### 3.4. HRV and Stress Assessment

Before exam, HRV did not differ among students who described themselves as stressed (64.4%) or not stressed (35.6%) prior to examination (*P* > 0.05 for all parameters, data not shown). However, during the 2 h of an exam, subjects who assessed themselves as not stressed (30%) displayed significantly lower HR (88.67 bpm, *P* = 0.013), higher SDNN (63.83 ms, *P* = 0.004), pNN50 (17.34%, *P* = 0.006), RMSSD (41.19 ms, *P* = 0.010), and SD1 (29.13 ms, *P* = 0.010), when compared to moderately stressed (55.6%) or highly stressed (14.4%) students. 

Moreover, two-way ANOVA showed no interaction effect between stress self-assessment levels and academic year (*P* > 0.05) before and during examination. However, during examination a significant interaction between gender and stress intensity assessment was observed, which was not found before the exam took place; further analysis also showed a significant correlation between stress intensity during examination and gender, where females reporting to be stressed are significantly more than males reporting the same stress intensity (females: 20% not stressed, 58.3% moderately stressed, 21.7% highly stressed; males: 50% not stressed, 50% moderately stressed; *P* = 0.002).

Thus, during examination there was consistency between self-assessment and the various physiological parameters of heart stress only in individuals who identified as not stressed. HRV in moderately stressed and highly stressed groups did not significantly differ during the examination (Table 4), which implies that the self-evaluation of stress levels remains a limitation. 

Box plot showing data distribution of HRV time domain parameters of n = 90 healthy individuals before (BE), during (DE), and after examination (AE), clustered based on gender. ○ and * represent outliers and extreme outliers respectively in the displayed data.

Box plot showing data distribution of HRV non-linear analysis parameters of n = 90 healthy individuals BE, DE, and AE, clustered based on gender. ○ and * represent outliers and extreme outliers respectively in the displayed data.

Box plot showing data distribution of HRV frequency domain parameters of n = 90 healthy individuals BE, DE, and AE, clustered based on gender. ○ and * represent outliers and extreme outliers respectively in the displayed data.

**Table 4:** HRV parameters measured during final exam over 2 h for 90 students grouped according to their feeling of being stressed during exam. Values presented as mean ± SEM.

	Not Stressed	Moderately Stressed	Highly Stressed	*P*-value	Significance	
HR [bpm]	88.67 ± 2.52	95.76 ± 1.42	98.26 ± 3.17	0.013		||
SDNN [ms]	63.83 ± 4.91	50.40 ± 1.95	47.65 ± 3.41	0.004	*	||
RMSSD [ms]	41.19 ± 4.78	29.34 ± 1.92	26.48 ± 3.48	0.010	*	||
pNN50 [%]	17.34 ± 3.17	8.49 ± 1.21	8.38 ± 2.67	0.006	*	||
LF [%]	38.06 ± 1.94	36.59 ± 1.41	35.08 ± 2.08	0.645		
HF [%]	18.67 ± 1.56	16.65 ± 1.18	15.52 ± 2.17	0.446		
LF/HF ratio	2.60 ± 0.30	2.80 ± 0.24	3.01 ± 0.57	0.772		
SD1 [ms]	29.13 ± 3.38	20.75 ± 1.35	18.73 ± 2.47	0.010	*	||
SD2 [ms]	85.14 ± 6.22	82.70 ± 15.41	64.56 ± 4.36	0.747		

*P*-value significance from ANOVA analysis: *, Not stressed vs. Moderately stressed; ||, Not stressed vs. Highly stressed.

### 3.5. HRV and Lifestyle Factors during Exam Period

Participants who had caffeine intake at an average of 1 cup per day (54.4%) during the night prior to exam or those who have the habit of smoking (22.2%), showed the same variability as those who avoided them. Similarly, of those who practiced a physical activity as a lifestyle (55.6%), physical activity did not have an impact during the examination time. Having breakfast before coming to the exam (65.5% of subjects) had no impact on the HRV before or during the exam (*P* > 0.05 for all parameters; data not shown).

## 4. Discussion

The objective of this study was to demonstrate the difference in HRV in three different phases of an exam among university students, where examination is considered a normal source of stress in the academic life at Lebanese University. Another goal was to compare, at a deeper level, the difference in HRV among students in different academic years and the existence of gender effect on stress levels; also, we aimed to assess the reliability of subjective feeling of stress before and during the examination. Moreover, lifestyle factors during examination period were studied as contributing factors for HRV changes, such as physical activity, nicotine use, and caffeine intake. To achieve these targets, participants were monitored for four hours covering one hour before their exam starts, two hours during their exam, and one hour after their exam was done under regular examination conditions.

Generally in examination period, students tend to have lower HRV compared to a non-exam period, with a decrease in parasympathetic activity of the heart, reflected by a decrease in RMSSD and increase in the sympathetic activity as shown by the increase in LF frequency parameter [7,8,16]. During the exam, students in our study presented the highest R-R interval and lowest HR compared to before and after the exam, which is opposite to that revealed in a previous study [13] where during examination, R-R interval was significantly the lowest. However, HRV in our study was the same before and during the exam, where it was significantly lower than that after the exam. Once the exam was over, SDNN, RMSSD and pNN50 significantly increased, indicating a recovery of the parasympathetic activity, and thus can be considered as a relaxation period as hypothesized. Our results reveal that HRV was reduced during mental stress conditions, that is before and during the examination and it augmented after that; whereas, another study revealed that only during examination, medical students presented a reduction in HRV compared to before and after the exam [17]. 

However, some contradictory results were obtained in certain studies, where no significant difference in SDNN was observed when comparing before and during an exam [12,14], nor in RMSSD when comparing an examination day to a normal academic day [8]. In all cases in our study, there was no significant change in the sympathetic to parasympathetic activity represented by the LF/HF ratio, similar to results revealed by previous investigations [8,12]. 

Surprisingly, there was no significant difference in HRV among students in different academic years of undergraduate studies, and therefore the same levels of stress were present. Students pursuing their first year of graduate program only showed lower HR and higher R-R compared to new students at the faculty (first year of their undergraduate studies) after the exam was over; this may reflect higher confidence in examination period once students start their second cycle studies. Carreras, and Fernandez-Castro showed that students in their second year of undergraduate studies showed significantly lower HR compared to students in their first year, explained as lower stress levels as a matter of adaptation to examination [18]. The present results at the Lebanese University can perhaps be attributed to the enrichment of courses with academic progression and thus the same levels of stress are detected regardless of the academic year and accommodation to exam procedures.

Several studies have reported that females present the same HR as males in the healthy state [19,20], while others debate whether females have higher HR than males in general conditions [19,21]. Our results show that males do possess lower HR in exam stress situation, and higher R-R intervals in accordance with several previous observations [19,22]. In addition, similar to what was observed in our study, men are proved to possess higher input of sympathetic activity of the nervous system, as shown by higher LF values compared to women in normal and stressful conditions [19,22,23]. This input is accompanied with higher LF/HF ratio, indicating a higher sympathetic input compared to parasympathetic input of the ANS in normal states [23]; however, in our results, there was no significant change in the LF/HF ratio except during the exam. Poincare plot short axis standard deviation, SD1 was significantly higher in men throughout the whole monitoring period. Overall, these data suggest that in real-life stress conditions, males do possess higher HRV and may control more their stress levels when concerned with their examination output when compared to females in the same frame of stress.

In a previous study, it was found that self-scored stressed individuals possess higher sympathetic activity, as observed by higher LF component and LF/HF ratio when compared to non-stressed group; this observation was detected under real-life stress condition [24]. Thus, another objective of our research was to study if we can rely on participant’s self-assessment of stress and if it can be correlated with changes in HRV among different groups before and during the occurrence of the stressful factor, which is university examination. Students were classified as to belonging to one of three groups based on their subjective feeling of being not stressed, moderately stressed, or highly stressed before and during the exam. During an exam, students would relief their stress and relax because of their ability to answer the given problems, or they would stress over when they had not prepared well. It was expected to see no significant change in HRV and thus stress before the exam started, which can be explained by overthinking about the possible exam questions and reviewing the course materials until the last minute before the exam. On the contrary, while the exam was taking place (2 h of exam), students belonging to different groups of self-assessed stress levels did show differences in HRV. Non-stressed students showed higher HRV; this is confirmed by those students having higher parasympathetic activity represented by enhanced values of RMSSD, pNN50 and SD1. 

Another aspect affecting HRV is the lifestyle of individuals, including physical activities and smoking habits. Studies have shown that a non-sedentary lifestyle, such as practicing yoga or running, helps recover HRV by adapting the heart to lower sympathetic and higher parasympathetic input of ANS [3,25]. In the current study, however, no significant change in HRV under mental stress conditions was observed between people who practiced some kind of activity and those who have a sedentary lifestyle; this may be attributed perhaps to the fact of low frequency practicing of the indicated activities. In the present study, the absence of significant change in HRV for subjects who drank caffeine before coming to the exam may be explained by the low amount of coffee intake, having an average of one cup in the previous 15 to 18 h. However, it has been reported that caffeine intake increases the HR, and thus decreases R-R and HRV; the sympathetic activity increases as shown by an increase in LF component in previous studies [5].

Future studies can be done to compare the effect of the mental stress of examination on students versus the stress experienced from attending the same class on a routine academic day. In addition, a broader sample survey can be assessed for lifestyle factors affecting the stress levels for routine life encountered events.

## 5. Conclusions

Overall, final examination time at the Lebanese University is considered a stressful period for students. The originality in our study is in following students for 2 h during their final exam. To our knowledge, there are no previous studies conducted based on our protocol where students are taking real exams. Stress levels seem to be the highest before and during the exam, indicated by a decrease in HRV, as opposed to the relaxation period after the exam. During the stress stimulant, components of the sympathetic nervous system increased as shown by an increase in LF and the LF/HF ratio, with a decrease in the values of parasympathetic activity as shown by a decrease in SDNN, RMSSD, pNN50 and SD1; these changes were reversed after the stressor was over, during relaxation time, when factors reflecting sympathetic activity decreased and a predominance of parasympathetic activity was observed. Of note, these results also demonstrate the consistency in the direction of change among the variables within our study. These stress levels are not well assessed by individuals prior to the stressor occurrence, as all students showed same degree of variability before the exam took place; however, while the exam is taking place, and while under the mental stress, individual assessment of stress can be relied upon, with students not feeling stressed showing higher variability in their heart beating pattern and lower sympathetic input compared to the self-assessed stressed group. Generally, in all conditions, females present higher HR and lower HRV compared to males, and thus are more prone to stress, even though males show higher sympathetic activity. It was surprising that no adaptation to the exam frame was observed at the level of HRV and stress among students in different academic years, although this may reflect the University’s curriculum design. Nonetheless, our study presents novel findings based on the real-time monitoring of a regular final examination of the university academic system.

## 6. Limitations of the Study

This study had some limitations concerning the recruitment of equal number of participants in different academic years, as well as the degree of gender equality; these factors could not be controlled due to the nature of voluntary participation. In addition, the use of one channel monitoring devices may have limited the sampling rate and filtering of ECG analysis. Obviously, another limitation of this study was the homogeneity in lifestyle (caffeine intake, smoking, and physical activity) of the enrolled participants.

## Figures and Tables

**Table 1:** Definition and indication of HRV parameters assessed in this study.

Time Domain	Frequency Domain	Non-Linear Domain
HR [bpm]: mean heart rate. SDNN [ms]: standard deviation of R-R. RMSSD [ms]: root mean square difference of successive R-R; reflects parasympathetic activity of the heart. pNN50 [%]: % of successive R-R interval that differ by more than 50 ms.	LF [%]: % of bands belonging to 0.04 to 0.15 Hz; reflects the sympathetic activity of the heart. HF [%]: % of bands belonging to 0.15 to 0.4 Hz; reflects the parasympathetic activity of the heart. LF/HF ratio: ratio of sympathetic to parasympathetic activity.	Poincare plot: SD1 [ms]: standard deviation along short axis, reflecting short-term HRV. SD2 [ms]: standard deviation along long axis reflecting long-term HRV.

**Table 2:** HRV parameters measured before, during and after final exam over 1 h 2 h and 1 h, respectively, for 90 students. Values presented as mean ± SEM.

	Before Exam (BE)	During Exam (DE)	After Exam (AE)	*P*-Value	Significance		
HR [bpm]	110.93 ± 1.74	93.99 ± 1.23	104.98 ± 1.53	<0.001	*	||	#
SDNN [ms]	72.60 ± 2.86	54.03 ± 1.99	89.78 ± 3.21	<0.001	*	||	#
RMSSD [ms]	40.68 ± 2.91	32.48 ± 1.93	46.55 ± 2.99	0.001			#
pNN50 [%]	9.09 ± 1.01	11.13 ± 1.29	13.01 ± 1.17	0.06		||	
LF [%]	21.87 ± 0.95	36.81 ± 1.02	20.00 ± 0.89	<0.001	*		#
HF [%]	10.24 ± 0.70	17.09 ± 0.86	9.76 ± 0.62	<0.001	*		#
LF/HF ratio	2.75 ± 0.17	2.77 ± 0.18	2.43 ± 0.12	0.246			
SD1 [ms]	28.30 ± 1.97	22.97 ± 1.37	32.66 ± 2.14	0.001			#
SD2 [ms]	96.82 ± 3.84	72.54 ± 2.57	122.14 ± 3.98	<0.001	*	||	#

*P*-value significance from ANOVA analysis: *, BE vs. DE; ||, BE vs. AE; #, DE vs. AE.

**Table 3:** Significance of HRV difference before, during, and after final exam over 1 h, 2 h and 1 h, respectively, between males (n = 30) and females (n = 60).

	Before Exam (BE)	During Exam (DE)	After Exam (AE)
HR [bpm]	0.050 *	0.018 *	<0.001 *
SDNN [ms]	0.170	0.056	0.059
RMSSD [ms]	0.009 ^||^	0.311	0.009 ^||^
pNN50 [%]	0.105	0.466	0.046 ^||^
LF [%]	0.007 ^||^	0.003 ^||^	0.022 ^||^
HF [%]	<0.001 ^||^	0.135	0.001 ^||^
LF/HF ratio	0.125	0.050 ^||^	0.280
SD1 [ms]	0.014 ^||^	0.310	0.008 ^||^
SD2 [ms]	0.173 ^||^	0.946	0.133 ^||^

* mean value for female significantly greater than male; || mean value for male significantly greater than female.

## Abbreviations

ANS	autonomic nervous system
AE	after exam
BE	before exam
DE	during exam
ECG	electrocardiogram
HR	heart rate
HRV	heart rate variability
